# Event and Comment

**Published:** 1919-02

**Authors:** 


### DENTAL REGISTER

THE MAGAZINE OF DENTISTRY

Vol. LXXIII FEBRUARY, 1919 No. 2

### EVENT AND COMMENT

**The Pulp Canal and Its Problem**

The agitation that was caused by the publication of fairly conclusive evidence, obtained by experimental research, has not yet subsided; neither has any well developed scheme of counteracting the influence of the pulpless tooth on systemic health been developed that meets the practical needs of the dental practitioner. Evidence is constantly accumulating of more or less serious systemic affection being brought under control by the eradication of diseased conditions associated with the teeth. The evidence is so convincing that all thoughtful dentists today are becoming more vigilant in ferreting out every possible open or hidden focus of disease associated with the teeth. In some cases the usual conservatism of the profession seems to be giving way to the most radical extermination of all suspiciously diseased teeth, and some go so far as to believe that no tooth from which the pulp has been, or is to be, removed, shall be left in the jaw. Much of the confusion is, of course, due to the fact that as yet we are not in possession of sufficient data to enable us to make a dogmatic declaration as to the proper procedure. There is, however, no doubt that some of our root canal treatments are so carelessly and unscientifically done, that unless nature was always on guard to protect the innocent, our profession in general could be seriously indicted for malpractice.

It is curious to note that in spite of the extensive discussions of this subject for the past two or three years some dentists still follow the old methods and seem willing to take chances which ordinarily they would condemn in other departments where no such hazards obtain. The practice of filling pulp canals is universal and dates historically back to the very beginning of conservative dentistry, and it seems that the practice has in a large measure been justified, even though crudely and unscientifically performed in most cases. In fact, it would seem that the more modern technical procedures have developed the dangerous characteristics of this practice. Can it be possible that in our efforts to completely sterilize and hermetically seal off the tooth from the environing tissues we are creating abnormal conditions that necessarily are not tolerable for the enveloping tissues of the tooth? Possibly we may by our methods interfere with nature's antitoxic methods.

We usually blame the reckless practice of sealing into the pulp canal antiseptic drugs of unknown ingredients and therapeutic values, as well as the overdosing of the apical space with cauterant and irritating poisons. We have gone to the limit of patient and skillful technical efforts to eradicate every possibility of the tooth becoming a source of infection, and to prolong its usefulness as a most valuable functional organ. It seems our efforts in this direction, because of the difficulties and expenditure of time involved, are becoming such a serious handicap that we are likely to prevent progress in this matter by our most careful technical endeavors.

The issue seems to be most critically drawn by our medical confreres, who can not conceive of our lack of willingness to co-operate with them on so vital a matter, even though it does involve the extraction of the teeth.

**Shall We Extirpate the Pulp?** While we are waiting to learn what our research scientists decide about this vexing problem, the sensible position for the dentist to take may be to follow the conservative methods of the past, especially when there are no positive contra indications, or when in doubt, use your best judgment. It might help us, perhaps, to work out a formula of our own as a basis from which we can formulate expeditiously a judgment in cases of emergency. How would something like the following do to refresh the memory and lessen the possibility of failure in some important part of our diagnosis:

We may extirpate the pulps and fill the root canals in deciduous teeth which it is desirable to retain in place to stimulate normal bone development until such time as the permanent tooth is ready to take the place of the deciduous tooth; providing there is no extensive necrosis of the bone and soft tissues about the root ends, and no hard cicatricial eschar will result from our therapeutic efforts to heal the abscess or necrosis. Owing to the general tendency of children's tissues to repair quickly, and the lack of liability to systemic infection we are justified in taking chances that would not be wise for patients in mature or aged life.

We can fill roots from which uninfected pulps are surgically removed under strictly aseptic conditions, provided the canals are immediately filled. This means that the canals are made easily accessible by convenient and ample approaches and that a complete and impervious filling can be inserted. In fact no root canal to which easy practicable approach that will permit adequate instrumentation can be filled with a sufficient degree of definiteness to justify the operation.

Pulps to which good access can be had that are only slightly infected, but not decomposed, may be extirpated by aseptic surgery and filled at once, provided the pulp remnants are thoroughly removed and no irritating disinfectants are forced through the apical foramina.

Pulps can safely be removed from teeth of most persons of average good health under the age of forty years, and if filled under careful aseptic instrumentation and with materials that will maintain antiseptic conditions, little chance of their becoming infective foci is probable. In patients beyond the age of forty there is liable to be more or less calcic deposits in the pulp canals, and the difficulties of thorough instrumentation are correspondingly increased. Such canals require so much time and effort to treat and fill them, and should acids be used for opening the canals, the probability that either infection or destructive chemicals will be forced into the apical space with ultimate fatal consequences.

In cases where particular teeth must be devitalized that they may be made serviceable for crown or bridge attachments, even for patients beyond the age of forty, there is no good reason why it may not be done successfully, providing the conditions of aseptic surgery are strictly complied with, and the tooth and patient are in reasonably good health. The risk in devitalizing such teeth is increased to a considerable degree because of the extra stress put upon such teeth, especially if used as abutments for bridges. There is a prevalent notion among dentists that devitalized teeth are more enduring than vital teeth, when used to support bridges. We never have been quite able to figure out why this should be so. We have seen many cases where vital teeth so utilized have quickly becomes diseased in the peridental tissues and had to be removed. The only way we can explain this phenomena is that owing to the normal functions being so closely related through the peridental tissues and the pulp organ, the primary injury to the gingival margin is directly referred to the pulp and the entire nerve and circulatory system becomes involved at the same time. This would cause excessive circulation and degenerative changes simultaneously. In the devitalized tooth there is no pulp inflammatory changes and even the peridental membrane has lost much of its natural irritability, so that the excessive strain on the tooth by the bridge does not cause irritation. If this were the case, devitalized teeth should be more promising for bridge abutment than vital teeth. It would not, however, justify careless devitalization of pulp and root canal fillings, for the reason that periapical infection would soon assert itself and lead to the loss of the abutment tooth. It would therefore seem that it is practicable as well as necessary to devitalize pulps for strategic reasons, but at the same time the operation must be of selected cases and should be done upon strictly aseptic and surgical principles.

It does not seem practicable to give up all efforts at devitalizing teeth because of the great number of useful reparations that are not possible by any other means that we know of at this time. There are some cases where root canal treatments of the kind under consideration here should not be encouraged, even though the conditions seem to demand them.

Teeth from which the pulps can not be successfully removed or devitalized, except by medicinal applications that are almost certain to leave periapical tissue that will become infected under the slightest provocation and in a short time lead to the loss of the tooth, or what may be worse, become a focus of infection for some distant tissue or vital organ. To this class of teeth belong most of the distal teeth and especially those having distal approximal cavities which make pulp extirpation almost impossible. The teeth of elderly patients in which the canals are not easily entered, and all contracted and irregularly formed canals, in fact, any canals that can not be entered and prepared for the root filling thoroughly and definitely. If it is impracticable to safely fill the canals of such teeth they had better be extracted at once, as they will assuredly make trouble sooner or later, and long before they give evidence of serious infection they have been prolific sources, perhaps, of insidious injury to the patient. Don't try to do the impracticable for the sake of a temporary advantage. It is no kindness to the patient; in fact it has been found to be the frequent cause of serious injury to many patients.

Another class of root canals that we should be more careful about putting under treatment, includes all pulpless teeth in which the infection has passed out of the canal and into the periapical tissues, particularly that class frequently classified as the blind abscess, and those in which the infected region is walled of as a granuloma. It is practicable in some cases to clear up these infected areas by medication through the root canal, but it generally results in a failure. They are more often eradicated by a thorough surgical currettement or apiectomy, which is applicable only to the teeth near the anterior part of the mouth, and very rarely to the distal molars. In like manner the so-called abscessed teeth with a discharging sinus, especially of long standing, is not a promising case even in cases where general systemic conditions seem favorable and in cases where infections are the rule, they are never favorable. Such teeth are proper cases for extraction in most cases, and when undertaken for special reasons the treatment must be the most thorough and skillful that is possible to warrant the risk.

The question is often asked as to what extent we are justified in removing the pulps from pyorrhetic teeth for the relief of sensitiveness, or to secure more thorough stability. Our experience in attempts to remove pulps from this class of teeth has not been wholly satisfactory. These pulp canals are almost always more or less calcified and generally it is impracticable to reach the apical region and remove all the pulp by a surgical operation. This is especially true of the molar teeth, and these are the teeth that usually call for some such treatment. These teeth ordinarily have no carious cavities to assist in making the approach to the pulp and an approach that is large enough to make the operation successful is difficult to make through the enamel and dentine, and we are always tempted to operate through a small opening that does not facilitate the operation or insure its success. In addition, we are likely to encounter crooked or restricted canals, which again render the operation complicated. But by patience and skillful manipulation, even these cases may be overcome and the results may justify the effort. The difficulties, however, make this a hazardous operation and a failure results so frequently that it may be classified as one of the operations that should not be undertaken except when an exceptional reason demands it.

In conclusion, we can only suggest that the operation of removing pulps from live teeth should not be undertaken except when clearly demanded, and then it should have the utmost care and skill possible. In the case of pulpless teeth with infected peridental areas the utmost care should be exercised in differentiating cases and only such as afford reasonable opportunity for surgical and therapeutic treatment, and have a reasonable promise of natural healing assistance, should be undertaken.

